# Evaluation of an mHealth App on Self-Management of Osteoporosis: Prospective Survey Study

**DOI:** 10.2196/53995

**Published:** 2024-04-01

**Authors:** Magnus Grønlund Bendtsen, Bodil Marie Thuesen Schönwandt, Mette Rubæk, Mette Friberg Hitz

**Affiliations:** 1 Research Unit, Medical Department, Zealand University Hospital Koege Denmark; 2 University of Southern Denmark Odense Denmark; 3 Institute of Clinical Medicine University of Copenhagen Copenhagen Denmark

**Keywords:** eHealth literacy, health literacy, mHealth, mobile health, eHealth, mobile health apps, self-management, osteoporosis, usability, acceptability

## Abstract

**Background:**

Mobile health (mHealth) technologies can be used for disease-specific self-management, and these technologies are experiencing rapid growth in the health care industry. They use mobile devices, specifically smartphone apps, to enhance and support medical and public health practices. In chronic disease management, the use of apps in the realm of mHealth holds the potential to improve health outcomes. This is also true for mHealth apps on osteoporosis, but the usage and patients’ experiences with these apps are underexplored.

**Objective:**

This prospective survey study aimed to investigate the eHealth literacy of Danish patients with osteoporosis, as well as the usability and acceptability of the app “My Bones.”

**Methods:**

Data on patient characteristics, disease knowledge, eHealth literacy, usability, and acceptability were collected using self-administered questionnaires at baseline, 2 months, and 6 months. The following validated questionnaires were used: eHealth Literacy Questionnaire, System Usability Scale, and Service User Technology Acceptability Questionnaire.

**Results:**

Mean scores for eHealth literacy ranged from 2.6 to 3.1, with SD ranging from 0.5 to 0.6 across the 7 domains. The mean (SD) System Usability Scale score was 74.7 (14.4), and the mean (SD) scores for domains 1, 2, and 6 of the Service User Technology Acceptability Questionnaire were 3.4 (1.2), 4.5 (1.1), 4.1 (1.2), respectively.

**Conclusions:**

Danish patients with osteoporosis are both motivated and capable of using digital health services. The app’s usability was acceptable, and it has the potential to reduce visits to general practitioner clinics, enhance health outcomes, and serve as a valuable addition to regular health or social care services.

## Introduction

### Background

Osteoporosis ranks as the fourth most prevalent chronic disease globally, carrying substantial negative personal and economic consequences [1]. Osteoporosis affects approximately 40% of women and 17% of men aged 50 years or older. Despite its high prevalence, osteoporosis is significantly underdiagnosed [2]. Although osteoporosis may remain asymptomatic for many individuals, it presents significant risks such as fractures, chronic pain, reduced daily activity, compromised quality of life, and increased mortality [1]. Lack of disease-specific knowledge among patients with osteoporosis is a global issue [3]. It has been shown that improved support for patients to understand osteoporosis upon diagnosis is required, along with support in self-management of the disease [4,5].

Mobile health (mHealth) technologies can be used for disease-specific self-management, and the technologies are experiencing rapid growth in the health care industry. These technologies use mobile devices, specifically smartphone apps, to enhance and support medical and public health practices [6]. Studies have demonstrated that, particularly in chronic disease management, the use of apps in the realm of mHealth holds the potential to enhance health outcomes [7]. Despite Slomian et al [8] addressing the potential of using mHealth apps to aid patients in self-managing osteoporosis in 2014, the implementation of such apps in the field of osteoporosis remains inadequate. A recently published systematic review and meta-analysis of digital health technologies for long-term self-management of osteoporosis identified 23 relevant apps for osteoporosis self-management and concluded that osteoporosis apps have the potential to support and improve the management of the disease. Furthermore, mHealth osteoporosis apps also appear to be valuable tools for patients and health care professionals. However, most of the identified apps that are currently available in the field of osteoporosis lack clinically validated evidence of their efficacy [9].

From 2015 to 2018, a team of researchers and health care professionals from Odense University Hospital, Denmark, developed and tested an mHealth app for women recently diagnosed with osteoporosis [10]. The development of the app was based on identified needs among patients and health care professionals [11,12]. The app evaluation revealed that patients perceived the app as providing confidence and reassurance, fostering equitable dialog during consultations, and offering readily accessible assistance for self-managing osteoporosis [13]. After the test period, the app was implemented at Odense University Hospital, Denmark. In 2019, the Danish Health Authority decided to support a nationwide rollout of the app, named “My Bones.” A group of health care professionals with expertise in osteoporosis were engaged in the app’s further development and testing. In September 2021, the app “My Bones” was launched as a freely available app on both the App Store and Google Play platforms. Visuals of the app are available in Multimedia Appendix 1.

After the app’s launch, the Research Unit of the Medical Department at Zealand University Hospital, Denmark, initiated an evaluation of the app focusing on usability, acceptability, eHealth literacy, and self-perceived knowledge of osteoporosis. The Consumer Health Information System Adoption Model was used as a theoretical framework, as a basis for the choice of questionnaires used in this study. In this model, eHealth literacy of the user, usefulness, and usability are all determining factors for the adoption of consumer health information systems like the app “My Bones.”

### Objective

The aim of this study was to investigate the eHealth literacy of Danish patients with osteoporosis and to assess the usability and acceptability of the app “My Bones.” Additionally, the aim was to assess the current level of disease knowledge within the patient group.

## Methods

### Study Design

This prospective survey study was conducted at Zealand University Hospital, Denmark, from January 2020 to February 2023. A total of 100 patients were recruited to test the app “My Bones.” At the time of the study, the app was owned and operated by OSAIA Health. The app provided patients with comprehensive information about osteoporosis, its risk factors, available treatment options, the diagnostic procedure for osteoporosis, and the process of a dual-energy x-ray absorptiometry (DXA) scan. Alongside this general information, the app provided guidance on maintaining a bone-healthy lifestyle, dietary requirements including calcium and vitamin D supplements, and recommendations for physical activity. App users also gained access to basic and safe training exercises tailored to their individual fracture risk and functional level, enabling them to engage in a foundational level of physical activity. The app includes 2 interactive modules. One of these was a calcium calculator that assists patients in measuring their daily calcium intake and determining whether they require calcium supplements. The second module was an interactive DXA graph that gives patients the option to plot their *T* score values for the spine and hip from each DXA scan.

Three self-administered questionnaires were developed and internally pretested before distribution at baseline (Q0), at 2 months (Q2), and at 6 months (Q6). The questionnaires are described under the Measures section. The results from the baseline (Q0) and 2-month (Q2) questionnaires are presented here. However, the results from the 6-month (Q6) questionnaire are excluded because of technical issues preventing data analysis.

### Sampling and Recruitment

Patients were recruited from the Outpatient Clinic of Endocrinology at Zealand University Hospital and through advertisements on the Danish Osteoporosis Association’s homepage and a Facebook page.

The inclusion criteria include patients diagnosed with osteoporosis based on either the *T* score criterion (*T*≤–2.5 at the lumbar spine, total hip, or hip neck) or a diagnostic osteoporotic fracture (fragility fracture of a vertebra with >20% compression or a hip fracture). Postmenopausal women or men over 45 years of age who can read and understand Danish and have access to, as well as the ability to use, a smartphone, tablet, or computer.

The exclusion criteria include patients with mental and cognitive conditions impairing their ability to use an app and read and understand questionnaire questions.

### Procedures

Patients with osteoporosis interested in participating were provided with both written patient-oriented materials and oral information about the study. Informed consent was obtained from the patients by the data controller. The signed consent forms were stored in a secure location, only accessible by the data controller. After signing the informed consent, participants were asked to indicate their preferred method of receiving the study questionnaires—either by email, through e-Boks (a digital postbox for communication between companies, public authorities, and private citizens), or in a printed copy. Detailed instructions on how to download the app from the App Store or Google Play were provided. If patients required assistance, the study staff aided in the process.

SurveyXact, a tool for creating electronic questionnaire-based surveys, was used to distribute the questionnaires to the patients and to establish a database. The data analyst gained access to the database only after all data had been collected and anonymized by way of a respondent key in the survey tool and transferred to the database. The survey tool is compliant with the European Union’s General Data Protection Regulation.

In cases of nonresponse, electronic reminders were dispatched, followed by telephone follow-ups. If participants still did not complete the questionnaire despite these attempts, they were classified as dropouts.

### Measures

#### Baseline Questionnaire (Q0)

Sociodemographic data include age, sex, education, occupation, time since diagnosis, and knowledge of the disease (self-reported *T* score of the spine and hip, medical treatment, and fracture history).

Data regarding education were initially reported on 6 levels and were subsequently consolidated into two levels for statistical analysis: (1) shorter education and (2) longer education. Longer education was defined as a bachelor’s degree or higher. Data concerning occupation were reported across 8 levels and were later condensed into two levels for statistical analysis: (1) currently working and (2) not currently working. The various education and occupation levels can be found in Table 1.

The eHealth Literacy Questionnaire (eHLQ) consists of 35 items representing 7 scales covering the eHealth Literacy Framework dimensions. Each scale has 4 to 6 items with a 4-point response option. Mean scores are calculated from each scale with equal weighting [14].

The International Physical Activity Questionnaire measures the amount of time an individual spends physically active during a normal 7-day week [15].

**Table 1:** Participant characteristics (N=95).

Characteristics		Participants
Age (years), mean (SD)		66.3 (8.4)
**Sex, n (%)**		
	Male	5 (5)
	Female	90 (95)
**Shorter education, n (%)**		42 (44)
	Primary school	8 (8)
	Vocational training, high school	21 (22)
	Higher education—short	12 (14)
**Longer education, n (%)**		53 (56)
	Higher education—intermediate	41 (43)
	Higher education—long	12 (13)
**Currently working, n (%)**		33 (35)
	Self-employed (professional)	3 (3)
	Civil servant	23 (24)
	Vocational	3 (3)
	Unskilled or semiskilled worker	4 (4)
**Not currently working, n (%)**		62 (65)
	Unemployed	3 (3)
	Early retirement	17 (18)
	Retirement pension	41 (43)
	Leave of absence	1 (1)

#### Two-Month Questionnaire (Q2)

The System Usability Scale (SUS) is a 10-item Likert scale questionnaire that offers a global perspective on subjective assessments of usability. The SUS generates a single number that represents a composite measure of the overall usability of the system under study, in this case, an app [16-18]. The questionnaire is validated in the Danish population.

The Service User Technology Acceptability Questionnaire (SUTAQ) is a questionnaire that consists of 22 items divided into 6 different subscales. For the purpose of this study, 3 of the 6 domains were included in the Q2 questionnaire. The included domains are (1) “Enhanced Care,” (2) “Increased Accessibility,” and (3) “Satisfaction.” The sum of each subscale indicates the degree of average internal agreement with it [19]. The domains “Privacy and Discomfort,” “Care Personnel Concerns,” and “Kit as a Substitution” were excluded because of their irrelevance resulting from the lack of health monitoring and interaction between patients and health care professionals via the app. The questionnaire is validated in the Danish population.

### Statistical Analysis

Statistical analysis was conducted using the SPSS statistical software (version 21; IBM Corp). Descriptive statistics were generated for participants’ characteristics and other dependent variables, with calculation of mean values and SD for normally distributed data and median with range for data not normally distributed. Frequency data were calculated for categorical data.

The relation between each of the 7 eHLQ domains and the covariates age, education, and occupation was investigated using a backward stepwise linear regression analysis.

The correlation between the SUS questionnaire score and age was investigated using the Pearson 2-tailed correlation analysis.

All statistical outcomes were examined against a *P* value of .05 to determine statistical significance. The study was not an intervention study, and no power calculation was performed.

### Ethical Considerations

The study was conducted in accordance with the principles of the Declaration of Helsinki. It received approval from the local Data Protection Authority (Reg-152-2020). Although the study did not meet the criteria that necessitate approval from the ethics committee, guidelines for obtaining informed consent were adhered to.

## Results

### Participant Characteristics

**Figure 1:**
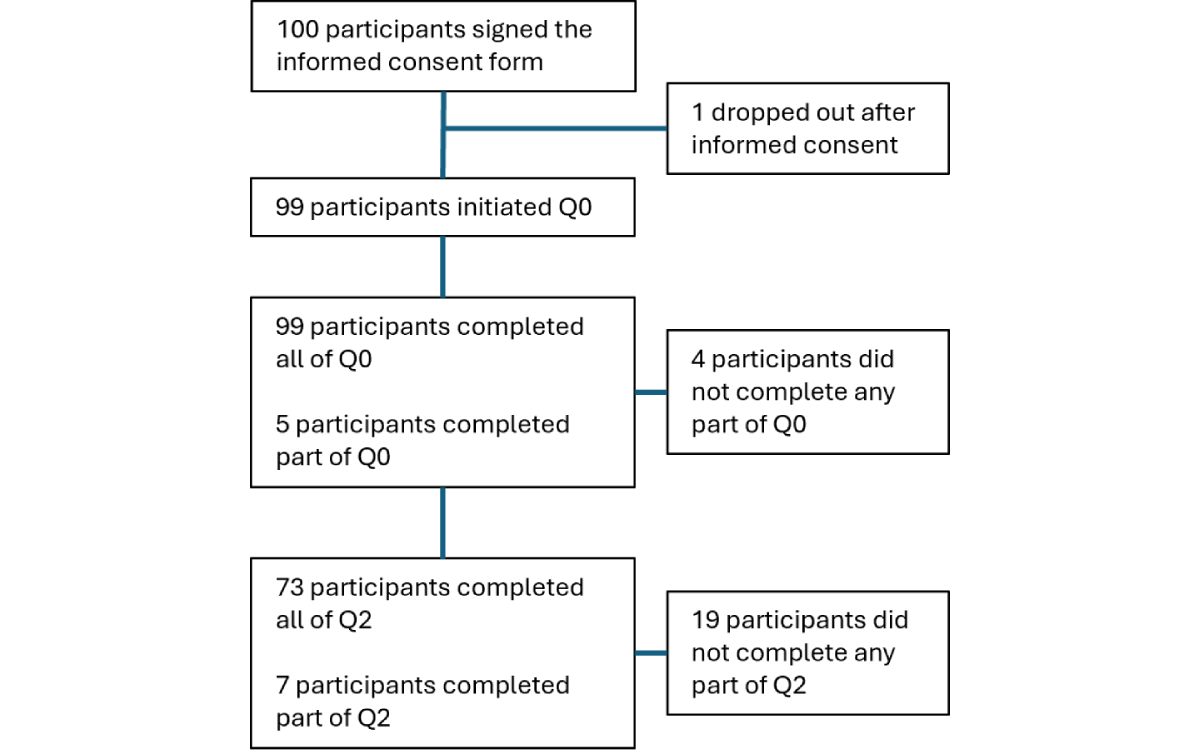
Participant flow diagram.

A total of 100 patients signed the informed consent. Of these, 95 responded to the entire or part of the baseline questionnaire (Q0), as shown in the flow diagram in Figure 1.

Of the 95 respondents, 90 were women and 5 were men. The mean (SD) age of the study population was 66.3 (8.4) years, and education levels were reported as shorter by 44% (42/95) of the participants and as longer by 56% (53/95) of the respondents. Regarding occupational status, 35% (33/95) stated a current connection to the labor market, while 65% (62/95) stated that they were either retired, unemployed, or on leave of absence (Table 1).

The mean time since the diagnosis of osteoporosis was 6 years (0-34 years). Among the 95 respondents, 52% (49/95) did not know the *T* score of the lumbar spine, and 68% (64/95) did not know the *T* score of the hip. Regarding self-reported osteoporosis status, 22% (21/95) and 4% (4/95) reported severe osteoporosis (*T* score<–3.0) in the spine region and the hip region, respectively (Table 2). Previous major osteoporotic fractures were reported by 49% (46/95) of the respondents (Table 2), while 67% (64/95) stated that they were currently undergoing medical treatment, the majority in treatment with bisphosphonate (Table 3).

Too much missing data hindered a meaningful analysis of the data from the International Physical Activity Questionnaire, and as a consequence, the results cannot be presented.

**Table 2:** *T* scores and fractures (N=95).

Characteristics			Participants
Time since diagnosis(years), mean (range)			6 (0-34)
***T*** **score lumbar spine, n (%)**			
	–2.5 to –3.0	24 (26)	
	–3.0 to –4.0	14 (15)	
	≥–4.0	7 (7)	
	Do not know my *T* score	49 (52)	
***T*** **score hip, n (%)**			
	–2.5 to –3.0	26 (28)	
	–3.0 to –4.0	3 (3)	
	≥–4.0	1 (1)	
	Do not know my *T* score	64 (68)	
**Previous fractures, n (%)**			46 (49)
	Wrist	21 (22)	
	Upper arm	3 (3)	
	Vertebrae	15 (16)	
	Hip	6 (6)	
	Other^a^	19 (20)	

^a^Other fractures reported: fracture of foot, ankle, heel, toe, finger, elbow, and tibia.

**Table 3:** Medication (N=93).

Characteristics			Participants
**Current treatment plan, n (%)**			64 (67)
	Oral BP^a^	16 (25)	
	IV^b^ BP	26 (41)	
	Denosumab	11 (17)	
	PTH^c^ analog	1 (2)	
	Romosozumab	2 (3)	
	Unknown to me	6 (9)	
	Other	2 (3)	

^a^BP: bisphosphonate.

^b^IV: intravenous.

^c^PTH: parathyroid hormone.

### eHLQ, SUS, and SUTAQ

A total of 90 participants completed the eHLQ section of the Q0 questionnaire. Mean (SD) scores reported ranged from 2.6 (0.5) to 3.1 (0.6) across the 7 domains (Table 4). A definition of each domain can be found in Multimedia Appendix 1.

A total of 79 participants responded to the SUS questionnaire with a mean (SD) score of 74.7 (14.4), while 74 participants responded to the SUTAQ, with mean (SD) scores for domains 1, 2, and 6 being 3.4 (1.2), 4.5 (1.1), and 4.1 (1.2), respectively (Table 5). Answers to individual questions of the SUTAQ are presented in Table 6.

The covariate “occupation” had an effect on eHLQ domain 4 (*P*=.04) with an adjusted *R*^2^ of 0.036 (Tables 7 and 8). No effects of the other covariates were found for the remaining domains of the eHLQ. No correlation was found between the SUS score and age (*P*=.37).

**Table 4:** eHealth Literacy Questionnaire (eHLQ, N=90).

Domain	Value, mean (SD)
eHLQ1: using technology to process health information	2.9 (0.6)
eHLQ2: understanding of health concepts and language	3.1 (0.5)
eHLQ3: ability to actively engage with digital services	3.0 (0.6)
eHLQ4: feel safe and in control	3.1 (0.5)
eHLQ5: motivated to engage with digital services	2.9 (0.6)
eHLQ6: access to digital services that work	2.9 (0.5)
eHLQ7: digital services that suit individual needs	2.6 (0.6)

**Table 5:** System Usability Score (SUS, n=80) and Service User Technology Acceptability Questionnaire (SUTAQ, n=73) scores at 2 months (Q2).

Questionnaire		Value, mean (SD)
SUS		74.6 (15.3)
**SUTAQ (scores from 1 to 6)**		
	Domain 1	4.1 (1.2)
	Domain 2	3.4 (1.1)
	Domain 3	4.5 (1.1)

**Table 6:** Service User Technology Acceptability Questionnaire individual statements (n=73).

Statement			Agreement with statement, n (%)								
			1^a^		2^a^	3^a^		4^a^	5^a^		6^a^
**Domain 1**											
	The kit has allowed me to be less concerned about my health status.	12 (16)		3 (4)		19 (26)	13 (18)		16 (22)	10 (14)	
	The kit has made me more actively involved in my health.	5 (7)		2 (3)		15 (21)	21 (29)		16 (22)	14 (19)	
	The kit allows the people looking after me, to better monitor me and my condition.	15 (21)		14 (19)		13 (18)	11 (15)		13 (18)	7 (10)	
	The kit can be or should be recommended to people in a similar condition to mine.	2 (3)		1 (1)		6 (8)	7 (10)		24 (33)	33 (45)	
	The kit can certainly be a good addition to my regular health or social care.	4 (5)		1 (1)		7 (10)	17 (23)		20 (27)	24 (33)	
**Domain 2**											
	The kit I received has saved me time in that I did not have to visit my GP clinic or other health or social care professional as often.	12 (16)		6 (8)		14 (19)	18 (25)		15 (21)	8 (11)	
	The kit I received has increased my access to care (health or social care professionals)	12 (16)		7 (10)		19 (26)	22 (30)		10 (14)	3 (4)	
	The kit I received has helped me to improve my health	6 (8)		7 (10)		13 (18)	25 (34)		16 (22)	6 (8)	
	The kit has made it easier to get in touch with health and social care professionals.	15 (21)		8 (11)		20 (27)	16 (22)		12 (16)	2 (3)	
**Domain 6**											
	The kit has been explained to me sufficiently.	5 (7)		10 (14)		12 (16)	12 (16)		15 (21)	19 (26)	
	The kit can be trusted to work appropriately.	1 (1)		1 (1)		5 (7)	11 (15)		32 (44)	23 (32)	
	I am satisfied with the kit I received.	2 (3)		3 (4)		12 (16)	7 (10)		28 (38)	21 (29)	

^a^1=strongly disagree; 2=moderately disagree; 3=mildly disagree; 4=mildly agree; 5=moderately agree; 6=strongly agree.

**Table 7:** Summary of regression analysis showing the relationship between occupation and eHealth Literacy Questionnaire domain 4.

Model	*R*	*R* ^2^	Adjusted *R*^2^	SE of the estimate
1	0.216^a^	0.047	0.036	0.5046

^a^Predictors: (Constant). Occupation.

**Table 8:** ANOVA^a^ for occupation’s effect on eHealth Literacy Questionnaire domain 4.

Model 1	Sum of squares	*df*	Mean square	*F* (*df*)	*P* value
Regression	1.099	1	1.099	4.315 (1,88)	.04^b^
Residual	22.405	88	0.255	—^c^	—
Total	23.504	89	—	—	—

^a^Dependent variable: eHealth Literacy Questionnaire domain 4.

^b^Predictors: (Constant). Occupation.

^c^Not available.

## Discussion

### Principal Results

Our analysis revealed eHLQ scores ranging between 2.6 and 3.1 across the 7 domains, with domain 7 “Digital services that suit individual needs” being the lowest-scoring domain and domains 2 “Understanding of health concepts and language” and 4 “Feel safe and in control” being the highest-scoring domains. These findings are comparable to those of another Danish study by Holt et al [20] on eHealth literacy conducted on 246 patients diagnosed with diabetes, other endocrine conditions, or gastrointestinal diseases. Holt et al [20] demonstrated eHLQ scores ranging between 2.6 and 3.1, with slightly lower scores across the 7 domains. This suggests that our findings are representative of Danish patients with chronic diseases. A Spanish study investigating electronic health literacy in 166 primary care patients, with a median age of 65 (52-78) years, revealed somewhat lower eHLQ scores ranging from 1.7 to 2.8 across the 7 domains [21]. An Australian study of 525 patients at 3 primary care clinics, with a mean age of 56.7, showed eHLQ scores ranging from 2.4 to 3.0 [22]. In comparison with participants from other countries, Danish patients seem to score higher on the eHLQ. These elevated scores among Danish individuals could be attributed to the extensive digitization of public services in Denmark. According to the latest report on digitalization in Denmark (2022), 95% of citizens reported receiving messages from public services through the digital mailbox known as “e-Boks.” Additionally, 74% of citizens aged 15 to 89 years use digital public services at least once a week, and 66% of citizens aged 16 to 74 years have booked a doctor’s appointment via the web and accessed health information through online sources [23]. On examining the eHLQ scores in this study, high scores are observed in domains 1-6, suggesting that Danish patients with osteoporosis are both motivated and able to use digital services related to health. The lowest-scoring domain, domain 7, indicates the current state of technology regarding digital services and points to a potential for developing more individualized services designed to meet the needs of patients.

The statistical analysis revealed a significant but relatively small effect of the covariate “occupation” on the scores of eHealth literacy domain 4 “Feel safe and in control.” This suggests that among the study sample, individuals who are currently working tend to feel safer and more in control when using electronic health services. However, given the small effect size within a limited sample, no definitive conclusions can be drawn. In the field of eHealth literacy, conflicting results have been reported regarding determinants of eHealth literacy scores. A recent systematic review demonstrated an association between eHealth literacy and age, sex, educational level, and family income [24]. Another study conducted by Arcury et al [25] failed to find a connection between eHealth literacy and sociodemographic factors. However, they did discover associations between eHealth literacy and the number of e-devices owned, as well as computer stress [25]. A related finding was reported by Richtering et al [26], who concluded that more time spent on the internet was associated with higher eHealth literacy among 453 patients with moderate to high cardiovascular risk. These findings suggest that factors other than sociodemographic variables play a role in eHealth literacy.

The app “My Bones” aims to assist patients in managing osteoporosis. To achieve this objective, it is crucial for the app to be both usable and well received by its users, prompting an investigation into its usability and acceptability. The analysis of the SUS questionnaire at the 2-month mark resulted in a mean (SD) score of 74.6 (15.3). According to Bangor et al [17], a score of approximately 70 indicates a user-friendliness rating of “Good.” Conversely, scores around 35 and 50 correspond to ratings of “Poor” and “OK,” respectively. On the other end of the spectrum, scores around 85 and 90 are associated with ratings of “Excellent” and “Best imaginable,” respectively [17]. Based on the SUS score, the usability of the “My Bones” app is considered acceptable.

The statistical analysis showed no correlation with age, contradicting the finding by Bangor et al that indicated a slightly negative correlation between the SUS score and age [27]. This discrepancy could be attributed to both the small sample size and the higher average age of this study population.

Analyzing the data from the 3 SUTAQ domains revealed a mean (SD) score of 4.1 (1.2) for domain 1 “Enhanced care,” which indicates a general agreement that the app “My Bones” can improve the care patients receive. The mean (SD) score of 4.5 (1.1) for domain 6 “Satisfaction” suggests acceptance and satisfaction with the app. However, the mean (SD) score of 3.4 (1.1) for domain 2 “Increased accessibility” indicates a lower level of agreement with beliefs that the app has facilitated the receipt of care from health care professionals.

In a Danish study involving 68 patients recruited from primary care and an outpatient clinic, the acceptability of a telehealth service was assessed using the SUTAQ. The scores obtained for domains 1, 2, and 6 were 5.0, 4.2, and 5.5, respectively [28]. Another Danish study focused on telehealth services for patients with chronic obstructive pulmonary disease, diabetes, and inflammatory bowel disease, and pregnant women with either diabetes or a need for enhanced care, demonstrated similar results. Scores for domain 1 ranged from 4.4 to 5.1, domain 2 from 3.8 to 4.7, and domain 6 from 5.2 to 5.6 [29]. Both studies exhibited higher scores across all 3 domains, indicating greater satisfaction and acceptance with the telehealth services compared with the “My Bones” app. One possible explanation for the lower scores in this study is that the SUTAQ was originally designed to assess the acceptability of telehealth services involving remote monitoring by health care professionals [19]. However, the app evaluated in our study does not function as a telehealth system, as it lacks monitoring, data collection by patients, and direct interaction with care personnel. Therefore, the applicability of SUTAQ results in describing the acceptability of the app among our study population may be limited. Certain statements within SUTAQ domains 1 and 2 are rendered irrelevant by the nature of the app. For instance, one statement in domain 2 referred to increased access to care through the received kit, which is not applicable because the app does not connect users with health care professionals. Similar issues were observed in other statements within these domains.

Nevertheless, it is worth noting that specific statements regarding the app’s usefulness showed promising results. Among the 74 participants who answered the SUTAQ questions, 56% agreed that the app saved them a visit to the general practitioner clinic. In total 65% agreed that the app helped them improve their health, 54% agreed that the app made them less concerned about their health status, and 70% agreed that the app made them more involved in their own health. Furthermore, 84% of participants agreed that the app can be a valuable addition to regular health or social care.

In summary, our findings from both the SUS and SUTAQ questionnaires underscore a high level of satisfaction with the “My Bones” app. While the usefulness of SUTAQ scores may be questionable, the responses to individual statements indicate that the app holds value for patients with osteoporosis.

### Other Findings

We discovered that over half of the 95 patients with osteoporosis had a significant gap in their knowledge about their condition, as they were not aware of their *T* scores. The *T* score reflects the severity of bone loss and risk of fractures. Knowing the *T* score can empower patients to take preventive measures in their daily lives to avoid injuries. Osteoporosis is a silent disease that often exhibits no symptoms; thus, the *T* score becomes a crucial indicator for patients to manage their condition. The fact that half of the patients lack awareness of the *T* score suggests a communication issue within the Danish health care system. Lack of disease-specific knowledge among patients with osteoporosis is a global issue [3], which might be addressed by apps like “My Bones.” However, because we did not inquire about the *T* score in our follow-up questionnaires, further research is needed to confirm whether the app can improve *T* score knowledge among patients with osteoporosis.

### Perspectives

Based on our findings, it appears that the app “My Bones” has the potential to effectively support the self-management of osteoporosis in this population as a supplement to current health care services. Further investigation should be undertaken to fully assess the app’s ability to enhance self-management among patients.

### Limitations

This study is subject to several limitations. First, the small population size restricts the generalizability of our findings. Additionally, individuals who are already accustomed to using smartphones and health management apps on a daily basis may have been more likely to participate, potentially introducing a bias toward higher eHealth literacy, acceptability, and satisfaction with the app. Recruitment primarily occurred at a single outpatient clinic and through online advertisements on the patient organization’s homepage and a Facebook page, introducing a sampling bias as most patients with osteoporosis in Denmark are typically treated at general practitioner clinics.

The use of questionnaires also presents limitations. Self-reported data on fractures, *T* scores, and medication are susceptible to response bias and may be less reliable than data obtained from registries. Conversely, the questions on *T* score and medication were specifically designed to provide insight into the patients’ understanding of their disease.

Despite these limitations, our study provides valuable insights into the eHealth literacy of patients with osteoporosis and the acceptability and usability of the first publicly available osteoporosis management app in Denmark.

### Conclusions

We uncovered a high level of eHealth literacy, indicating that Danish patients with osteoporosis possess both the motivation and ability to use mHealth services. The app demonstrated acceptable usability and garnered general satisfaction among users. These findings bolster the viability of an app for self-management of osteoporosis to support Danish patients. The app holds the potential to reduce visits to the general practitioner clinic, enhance health outcomes, and serve as a valuable addition to regular health or social care. However, further investigation is necessary to thoroughly evaluate its effectiveness in improving self-management of osteoporosis.

## Appendix

Multimedia Appendix 1 Scale names and construct definitions of the eHealth Literacy Questionnaire (eHLQ) and visuals of the app “My Bones”.

## Abbreviations

DXA: dual-energy x-ray absorptiometry
eHLF: eHealth Literacy Framework
eHLQ: eHealth Literacy Questionnaire
IPAQ: International Physical Activity Questionnaire
mHealth: mobile health
SUS: System Usability Scale
SUTAQ: Service User Technology Acceptability Questionnaire
