# Supplementary material for: Evaluation of an mHealth App on Self-Management of Osteoporosis: Prospective Survey Study
Source: Interact J Med Res. 2024 Apr 1;13:e53995. doi: 10.2196/53995 (PMC11019424; doi:10.2196/53995)
Supplement: Multimedia Appendix 1 [file ijmr_v13i1e53995_app1.docx]

**Multimedia Appendix 1**

Scale names and construct definitions of eHealth Literacy Questionnaire (eHLQ)

| **No.** | **Domain name** | **Domain definition** |
| --- | --- | --- |
| 1 | Using technology to process health information  (5 items) | Able to read, write, and remember, apply basic numerical concepts, and understand context-specific language (e.g. health, technology or English) as well as critically appraise information. Know when, how and what information to use. |
| 2 | Understanding of health concepts and language  (5 items) | Know about basic physiological functions and own current health status. Aware of risk factors and how to avoid them or reduce their influence on own health as well as navigating the health care system. |
| 3 | Ability to actively engage with digital services  (5 items) | Being comfortable using digital services for handling information. |
| 4 | Feel safe and in control  (5 items) | Feel that they have the ownership of personal data stored in the systems and that the data are safe and can be accessed only by people to whom they are relevant (such as own doctor and nurse). |
| 5 | Motivated to engage with digital services  (5 items) | Feel that engaging in the use of digital services will be useful for them in managing their health. |
| 6 | Access to digital services that work  (6 items) | Have access to digital services that the users trust to be working when they need it and as they expect it to work. |
| 7 | Digital services that suit individual needs  (4 items) | Have access to digital services that suit the specific needs and preferences of the users. This includes responsive features of both the information technology and health care system as well as adaptation of devices and interfaces to be used by people with physical and mental disabilities. |

Visuals of the app ‘My Bones’

| ^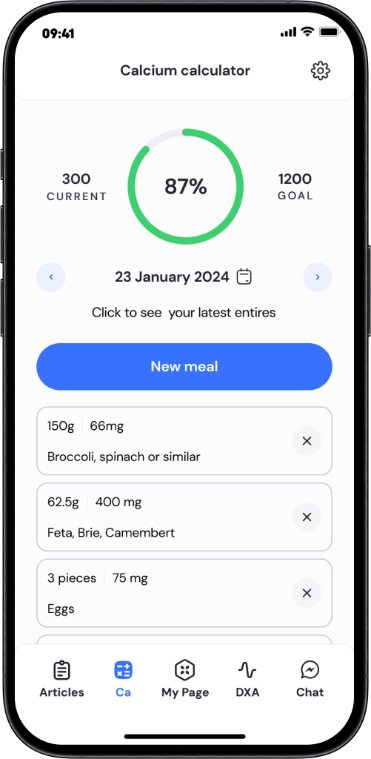^  Calcium calculator | ^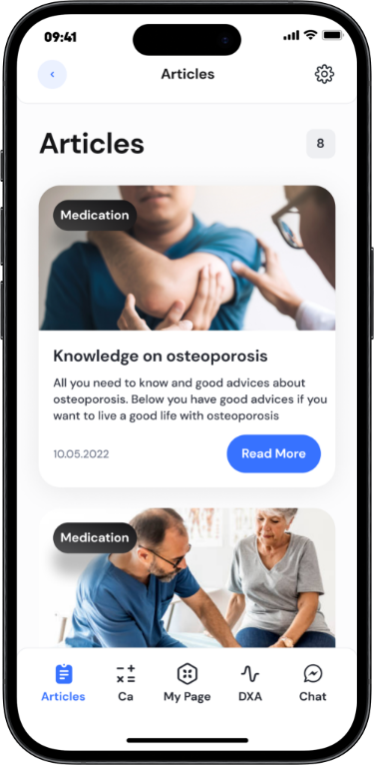^  General information on osteoporosis,  diagnosis, and treatment |
| --- | --- |
| 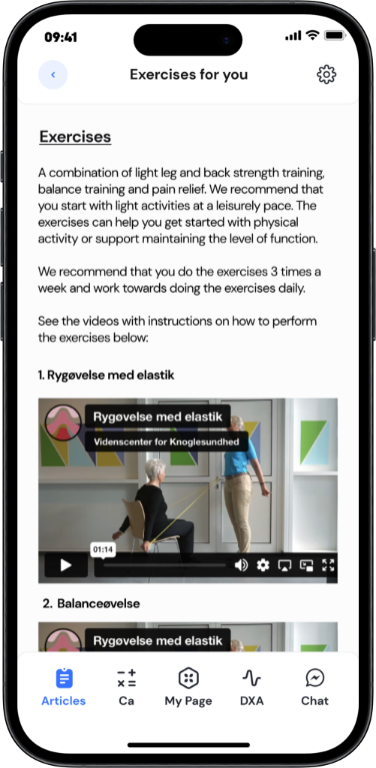  Videos on basic exercises for patients  with osteoporosis | 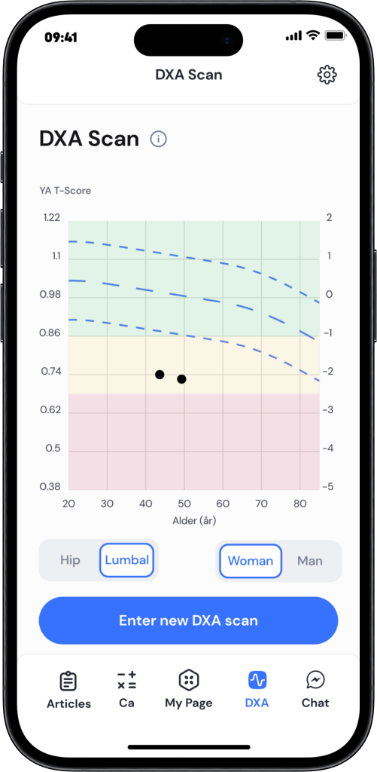  Overview of T-score values  from DXA scans |
